# AR ubiquitination induced by the curcumin analog suppresses growth of temozolomide-resistant glioblastoma through disrupting GPX4-Mediated redox homeostasis

**DOI:** 10.1016/j.redox.2019.101413

**Published:** 2019-12-26

**Authors:** Tzu-Chi Chen, Jian-Ying Chuang, Chiung-Yuan Ko, Tzu-Jen Kao, Pei-Yu Yang, Chun-Hui Yu, Ming-Sheng Liu, Siou-Lian Hu, Yu-Ting Tsai, Hardy Chan, Wen-Chang Chang, Tsung-I. Hsu

**Affiliations:** a Allianz Pharmascience Limited, Taipei, Taiwan; b Graduate Institute of Neural Regenerative Medicine, College of Medical Science and Technology, Taipei Medical University, Taipei, Taiwan; c Ph.D. Program for Neural Regenerative Medicine, College of Medical Science and Technology, Taipei Medical University and National Health Research Institutes, Taipei, Taiwan; d TMU Research Center of Neuroscience, Taipei Medical University, Taipei, Taiwan; e TMU Research Center of Cancer Translational Medicine, Taipei Medical University, Taipei, Taiwan; f Cell Physiology and Molecular Image Research Center, Wan Fang Hospital, Taipei Medical University, Taiwan; g National Institute of Cancer Research, National Health Research Institutes, Taiwan; h Graduate Institute of Medical Sciences, College of Medicine, Taipei Medical University, Taipei, Taiwan

**Keywords:** AR, ALZ003, GPX4, Glioblastoma

## Abstract

Drug resistance is the main obstacle in the improvement of chemotherapeutic efficacy in glioblastoma. Previously, we showed that dehydroepiandrosterone (DHEA), one kind of androgen/neurosteroid, potentiates glioblastoma to acquire resistance through attenuating DNA damage. Androgen receptor (AR) activated by DHEA or other types of androgen was reported to promote drug resistance in prostate cancer. However, in DHEA-enriched microenvironment, the role of AR in acquiring resistance of glioblastoma remains unknown. In this study, we found that AR expression is significantly correlated with poor prognosis, and AR obviously induced the resistance to temozolomide (TMZ) treatment. Herein, we observed that ALZ003, a curcumin analog, induces FBXL2-mediated AR ubiquitination, leading to degradation. Importantly, ALZ003 significantly inhibited the survival of TMZ-sensitive and –resistant glioblastoma *in vitro* and *in vivo*. The accumulation of reactive oxygen species (ROS), lipid peroxidation and suppression of glutathione peroxidase (GPX) 4, which are characteristics of ferroptosis, were observed in glioblastoma cell after treatment of ALZ003. Furthermore, overexpression of AR prevented ferroptosis in the presence of GPX4. To evaluate the therapeutic effect *in vivo*, we transplanted TMZ-sensitive or -resistant U87MG cells into mouse brain followed by intravenous administration with ALZ003. In addition to inhibiting the growth of glioblastoma, ALZ003 significantly extended the survival period of transplanted mice, and significantly decreased AR expression in the tumor area. Taken together, AR potentiates TMZ resistance for glioblastoma, and ALZ003-mediated AR ubiquitination might open a new insight into therapeutic strategy for TMZ resistant glioblastoma.

## Highlights

• ALZ003, the curcumin analog, inhibits TMZ–resistant glioblastoma.
• ALZ003 induces AR degradation through FBXL2-mediated ubiquitination.
• ALZ003 induces ferroptosis through blocking AR-mediated GPX4 expression.
• ALZ003 increases ROS and lipid peroxidation through reducing GPX4 expression.

## Graphical abstract

**Figure:**
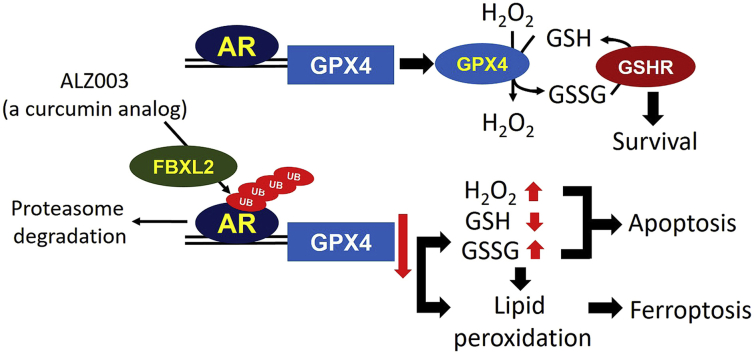

## 1 Introduction

Resistance to therapeutic treatment is the main reason causing death of cancer patients, especially of glioblastoma. The standard care protocol for glioblastoma is temozolomide (TMZ)-mediated chemotherapy conjugated with radiotherapy [1]. The tumor-suppressive effect of TMZ is restricted within a short window caused by the high frequency of recurrence in glioblastoma. Besides, it's difficult to identify a potent compound against brain tumors due to low efficiency of blood-brain-barrier (BBB)-crossing and high side effects on normal brain cells. TMZ is still a first-line chemotherapeutic drug for glioblastoma even though resistance is always identified after treatment. The discovery of a novel compound, which exhibits high BBB-crossing rate, potent tumor-suppressive effect and safety for normal brain cells, will highly improve the quality of intervention for glioblastoma.

Androgen receptor (AR), a steroid hormone receptor, mediates physiological functions through binding to its steroid ligands including dehydroepiandrosterone (DHEA), testosterone and dihydrotestosterone [[2], [3], [4], [5]]. Particularly, after ligand-induced conformational change, AR dissociates from heat shock protein and moves from cytoplasm to nucleus for DNA transcription [[2], [3], [4]]. Alternately, AR can be activated independently of androgens. Phosphorylation on multiple tyrosine residues restores AR function in androgen-deficient condition [6]. SRC and Her2/neu were shown to activate AR in the absence of androgens [6]. In addition, AR is a well-recognized biomarker to predict prognosis in prostate cancer, and acquired ligand-independent activity of AR is a predictor for drug-resistant prostate cancer [2]. Moreover, dysregulations of AR activity and expression have been reported in various types of cancer, such as bladder [3], ovarian [7], breast [4] and salivary gland cancers [5]. However, in glioblastoma development which relies on the production of neurosteroids, the role of AR still remains unclear. Importantly, we previously confirmed that DHEA, the ligand of AR, promotes acquiring resistance of glioblastoma in response to TMZ [8], further driving us to elucidate whether AR is a promising target to overcome drug resistance. Herein, we attempt to understand whether AR regulates the survival of glioblastoma, and to clarify whether a potential compound targeting AR is effective to treat glioblastoma.

Curcumin is a well-known tumor suppressive compound, and is considered to exhibit therapeutic effect on neurodegenerative disease because of its ability to cross blood-brain-barrier [9,10]. In the past decade, curcumin was shown to inhibit glioblastoma through suppressing multiple oncogenic signaling [[11], [12], [13]]. However, rapid systemic elimination limits the anti-tumor effect of curcumin [14]. Herein, we have developed ALZ003, which is structurally analogous to curcumin with the property of degradation of AR protein [15]. Importantly, FDA has granted Orphan Drug Designation to ALZ003 for the treatment of glioblastoma recently. In the present study, we showed that ALZ003 significantly inhibited TMZ-sensitive and –resistant glioblastoma *in vitro* and *in vivo*, whereas it exhibited non-cytotoxic effect on normal astrocytes. Moreover, ALZ003 downregulated glutathione peroxidase 4 (GPX4) expression, leading to lipid peroxidation and abundant ROS accumulation. As caspases activation and lipid peroxidation are known to induce apoptosis and ferroptosis, respectively, ALZ003 is a potential therapeutic drug for primary and recurrent glioblastoma.

## 2 Materials and methods

### 2.1 Bioinformatics analysis

**Fig. 1:**
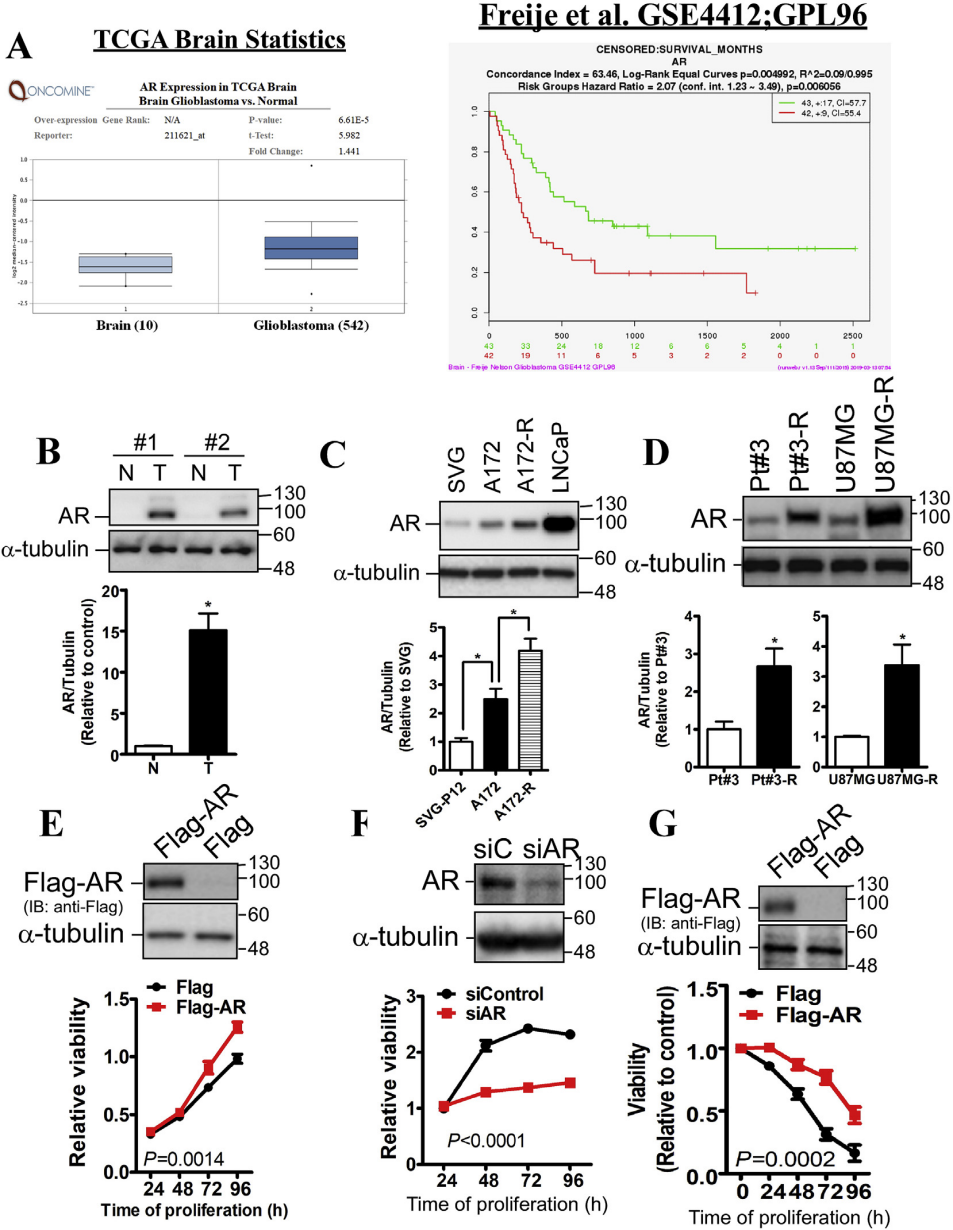
**The correlation of AR expression with prognosis and drug resistance in glioblastoma.** A. Bioinformatics analysis for AR in glioblastoma. The mRNA expression of AR in normal brain and glioblastoma tissues was compared in the TCGA dataset using the *Oncomine* website. The correlation of AR mRNA expression with prognosis was acquired from GSE4412 released by Freije et al. using the *SurvExpress* website. *P*-value was indicated. B. The protein level of AR in human specimens was analyzed using western blotting. N: normal brain tissue; T: glioblastoma tissue. The results of two paired specimens were quantitated. Data were expressed as mean ± s.e.m. (*p < 0.05). C, D. The expression of AR in multiple cell glioblastoma cell lines and a prostate cancer cell line, LNCaP (positive control). SVG p12 (SVG) is an immortalized fetal glial cell line. Experiments were performed three times independently, and data were expressed as mean ± s.e.m. (*p < 0.05). E. After transfection with Flag-AR or siRNA-mediated AR knockdown (F) for 2 days, U87MG-R cells viability was estimated by MTT assay. G. After transfection with Flag-AR for 24 h, U87MG cells were treated with 100 μM TMZ for the indicated interval. Viability was estimated by MTT assay. Experiments were performed three times independently, and data were expressed as mean ± s.e.m. The significant difference between control and knockdown/overexpression groups was analyzed using two-way ANOVA. *P*-value was indicated. The cell lysates were subjected to western blotting to confirm Flag-AR expression and AR knockdown.

The comparison of AR mRNA level between normal and glioblastoma was performed using the *Oncomine* website (https://www.oncomine.org/resource/login.html), and TCGA lower grade glioma dataset was analyze as shown in Fig. 1A. Other 7 glioma datasets of *Oncomine* were also analyzed as shown in Supplementary Table S1. The correlation of AR mRNA level with glioblastoma patient's prognosis was analyzed using the *SurvExpress* website (http://bioinformatica.mty.itesm.mx:8080/Biomatec/SurvivaX.jsp) [16]. Particularly, for Fig. 1B, the glioma dataset released by Freije et al. [17] was selected for analysis.

### 2.2 Cell lines, chemical compounds, and siRNA

U87MG and A172 cells were purchased from ATCC (Manassas, VA, USA) and maintained in DMEM containing 10% fetal bovine serum (FBS), 100 μg/ml streptomycin and 100 μg/ml penicillin G (Thermo Fisher Scientific, Waltham, MA, USA). Luciferase-expressed U87MG cells were provided by Dr. Kwang-Yu Chang (National Health Research Institutes, Tainan, Taiwan), and cells were maintained in DMEM containing 10% FBS, 100 μg/ml streptomycin and 100 μg/ml penicillin G. TMZ-resistant cells were established as described previously [18,19]. TMZ was purchased from MilliporeSigma Corporate (St. Louis, MO, USA). ALZ003 compound was provided by Allianz Pharmascience Limited (Taipei, Taiwan). Both of TMZ and ALZ003 were dissolved in dimethyl sulfoxide (DMSO, MilliporeSigma Corporate), and stored at −20 °C. siRNA targeting AR or GPX4 was purchased from GE Healthcare Dharmacon, Inc. (Lafayette, CO, USA).

### 2.3 Primary glioblastoma cells, Pt#3

The use of human specimens was approval by the Institute Review Board (IRB)/Ethics Committee from the office of human research in Taipei Medical University (Taipei, Taiwan). The patient consent and isolation protocol were described previously [18]. The culture condition of Pt#3 cells was the same with that of other glioblastoma cell lines.

### 2.4 Isolation and culture of mouse primary astrocytes

Brain cortex was excised from post-natal Day 0 mice, and homogenized by 10 U/ml trypsin at 37 °C for 30 min. After mixing with DMEM containing 10% FBS (Thermo Fisher Scientific, Waltham, MA, USA), the cell suspension was filtered by a 70-μm nylon mesh filter (Small Parts, Inc., Florida, MI, USA). Cells were seeded onto culture plates at a density of 0.5 × 10^6^ cells/cm^2^, and cultured in DMEM containing 10% FBS. After maintaining 72 h followed by the removing suspended cells, adhered cells were used for further experiments until 4th passage.

#### 2.4.1 3-(4,5-Dimethylthiazol-2-yl)-2,5-diphenyltetrazolium bromide (MTT) assay

The experimental procedure followed the protocol as described previously [8,18]. Briefly, after treatment, cells were incubated with the medium containing 0.5 mg/ml of MTT reagent for 1 h at 37 °C. Subsequently, MTT-containing medium was removed, and cells were incubated with 200 μl of DMSO for 30 min at room temperature to dissolved formazan crystals. Microplate Absorbance Reader (Bio-Rad Laboratories, Inc., Hercules, CA, USA) was used to estimate the absorbance of DMSO extracts at 570 nm wavelength.

#### 2.4.2 Immunoprecipitation

The protocol of previous study was followed. Cells were harvested in protein lysis buffer containing protease inhibitor cocktail (MerckMillipore, Bedford, MA, USA). Five hundred microgram of protein was incubated with the anti-AR (abcam, Cambridge, UK) or anti-ubiquitin (MerckMillipore) antibody followed by the precipitation using protein A/G agarose (MerckMillipore). After washing 4 times by lysis buffer, the complex was mixed with 2X SDS sample buffer containing β-mercapto-ethanol (MilliporeSigma Corporate), and subjected to western blotting.

### 2.5 Western blotting

After electrophoresis, proteins on the SDS-PAGE were transferred to polyvinylidene difluoride membrane and blocked by 5% non-fat milk. Blocked membrane was incubated with the primary antibody overnight at 4 °C followed by incubating with the secondary antibody against rabbit or mouse IgG for 1 h. After mixing with ECL chemiluminescent substrates, (GE Healthcare Life Sciences) were captured using the ChemiDoc™ Touch Imaging System (Bio-Rad Laboratories, Inc.). Primary antibodies were listed in the Supplementary Table S2.

### 2.6 Caspase 3/7, 8, 9 detection

Caspase-Glo® 3/7, 8 and 9 assay kits were purchased from Promega Inc. (Fitchburg, WI, USA), and were used according to the manufacturer instructions. After treatment, the cultured medium was collected, and mixed with reagents provided by Caspase-Glo® assay kit. The signal representing caspases was detected by the luminometer (Promega Inc.).

### 2.7 ROS analysis

Cellular ROS was estimated using dihydrorhodamine 123 (DHR, Thermo Fisher Scientific). After incubation with DHR for 30 min at 37 °C, DHR-derived fluorescence was measured by the flow cytometry and analyzed by the GuavaSoft software (MerckMillipore).

### 2.8 H_2_O_2_ and GSH/GSSG analysis

The H_2_O_2_-Glo® (Catalog #G8821) and GSH/GSSG-Glo® (Catalog #V6612) assay kits were purchased from Promega Inc. and used according to the manufacturer instructions. For these 2 experiments, cells were seeded into 96-well plates. To detect H_2_O_2_ and evaluate GSH/GSSG, cell medium and lysates were prepared, respectively. After mixing with reagents provided in the assay kit, concentration of H_2_O_2_ and GSH in the mixture was determined by the luminometer (Promega Inc.). GSSG and the ratio of GSH/GSSG were calculated according to the instruction.

### 2.9 Glutathione reductase (GSHR) activity analysis

The assay kit for GSHR activity was purchased from Biovision Inc. (Catalog #K761-200; Milpitas, CA, USA), and used according to the manufacturer instructions. Cells (1 × 10^6^) were lysed for centrifugation, and supernatant was mixed with assay buffers provided in the kit. The absorbance of mixture was determined by ELISA reader (Bio-Rad Laboratories, Inc.) at OD 405 nm.

### 2.10 Lipid peroxidation

BODIPY™ 581/591C11 (Thermo Fisher Scientific), a commercial lipid peroxidation sensor, was used according to the manufacturer instructions. After drug treatment and siRNA-mediated gene knockdown, cells in the dishes were stained by adding BODIPY reagent and DAPI staining solution (Catalog # ab228549, abcam) into media. After 30 min, cells were monitored under fluorescence microscope (Leica).

### 2.11 Animal experiments

NOD-SCID male mice (8-week-old) were purchased from BioLASCO Taiwan Co., Ltd. (Taipei, Taiwan), and maintained at the National Health Research Institutes (Tainan, Taiwan). For intracranial transplantation, experimental procedure followed the previous study [18], and was performed under sterile condition. For glioblastoma and TMZ-resistant glioblastoma transplantation, luciferase-expressed U87MG cells (2 × 10^5^) and U87MG-R cells (2 × 10^5^) were injected into the cortex, respectively, at the depth of 3 mm using stereotactic guidance and microprocessor single syringe (Harvard Apparatus, Holliston, MA, USA). After 10 days of transplantation, TMZ (15 mg/kg) [18] and ALZ003 were orally and intravenously administrated three times per week, respectively. The luciferase activity of U87MG was monitored by IVIS 200 system (Xenogen Corporation, Alameda, CA, USA). Particularly, to prepare the formulation for intravenous injection, ALZ003 stock in DMSO (50 mg/ml) was diluted in the mixture of PBS and Tween 80 (MilliporeSigma Corporate). The formulation for injection was prepared 30 min before injection, and was not stored for other experiments.

### 2.12 Immunohistochemistry (IHC)

The protocol published previously was followed [18]. The whole brain was embedded in paraffin, and 5-μm slices were prepared. IHC staining was performing using VECTASTAIN® ABC AP Kits (Vector Laboratories, Inc., Burlingame, CA, USA). IHC Score was defined as Score 4: strong expression; Score 3: moderate; Score 2: weak; Score 1: negative expression in the tumor part or basal expression on the brain without obvious tumors.

#### 2.12.1 Reverse transcription-quantitative polymerase chain reaction (RT-qPCR)

Total RNA was extracted using RNA Isolation Kit (Zymo Research, Irvine, CA, USA). After reverse transcription (Thermo Fisher Scientific), the mixture containing 1 μg of cDNA and SYBR Green (Thermo Fisher Scientific) was subjected to qPCR by PCR machine (Thermo Fisher Scientific) using primers targeting AR: F: CGGAAGCTGAAGAAACTTGG; R: ATGGCTTCCAGGACATTCAG.

### 2.13 Statistical analysis

The difference between 2 groups was analyzed by Student's *t*-test. To compare the difference in effect of TMZ with multiple doses on viability between control group and knockdown/overexpression group (Fig. 1E, F, G), two-way analysis of variance (ANOVA) was performed. To compare the luciferase activity in multiple groups at multiple time points (Fig. 7C), two-way ANOVA was used. For survival analysis (Fig. 7, Fig. 8A), the log-rank test was performed. *P*-value < 0.05 was considered as the significant difference.

## 3 Results

### 3.1 AR overexpression contributes to poor prognosis and TMZ resistance

To understand the clinical relevance of AR in glioblastoma, we analyzed the correlation between AR expression and prognosis using *Oncomine* and *SurvExpress*. Accordingly, AR expression was significantly increased in glioblastoma compared with normal brain tissue, and high AR expression significantly correlated with shorter survival period (Fig. 1A and Supplementary Table S1). In consistence with bioinformatics analysis, AR expression was obviously increased in human glioblastoma specimens determined by western blotting analysis (Fig. 1B). In addition, we also found that AR expression was further increased in TMZ-resistant glioblastoma cells, A172-R, U87MG-R and Pt#3-R, all of which were established in the previous studies [8,18,19] (Fig. 1C and D). Based on these investigation, we hypothesize that AR promotes glioblastoma growth and induces drug resistance. Therefore, we investigated effect of AR on proliferation and cellular sensitivity responded to TMZ. As shown in Fig. 1E and F, Flag-AR overexpression significantly increased proliferation whereas knockdown of AR significantly inhibited proliferation of U87MG-R cells. Moreover, overexpression of AR significantly increased the tolerance of U87MG cells in response to TMZ, leading to TMZ resistance (Fig. 1G).

### 3.2 ALZ003 inhibits glioblastoma survival through decreasing AR expression

**Fig. 2:**
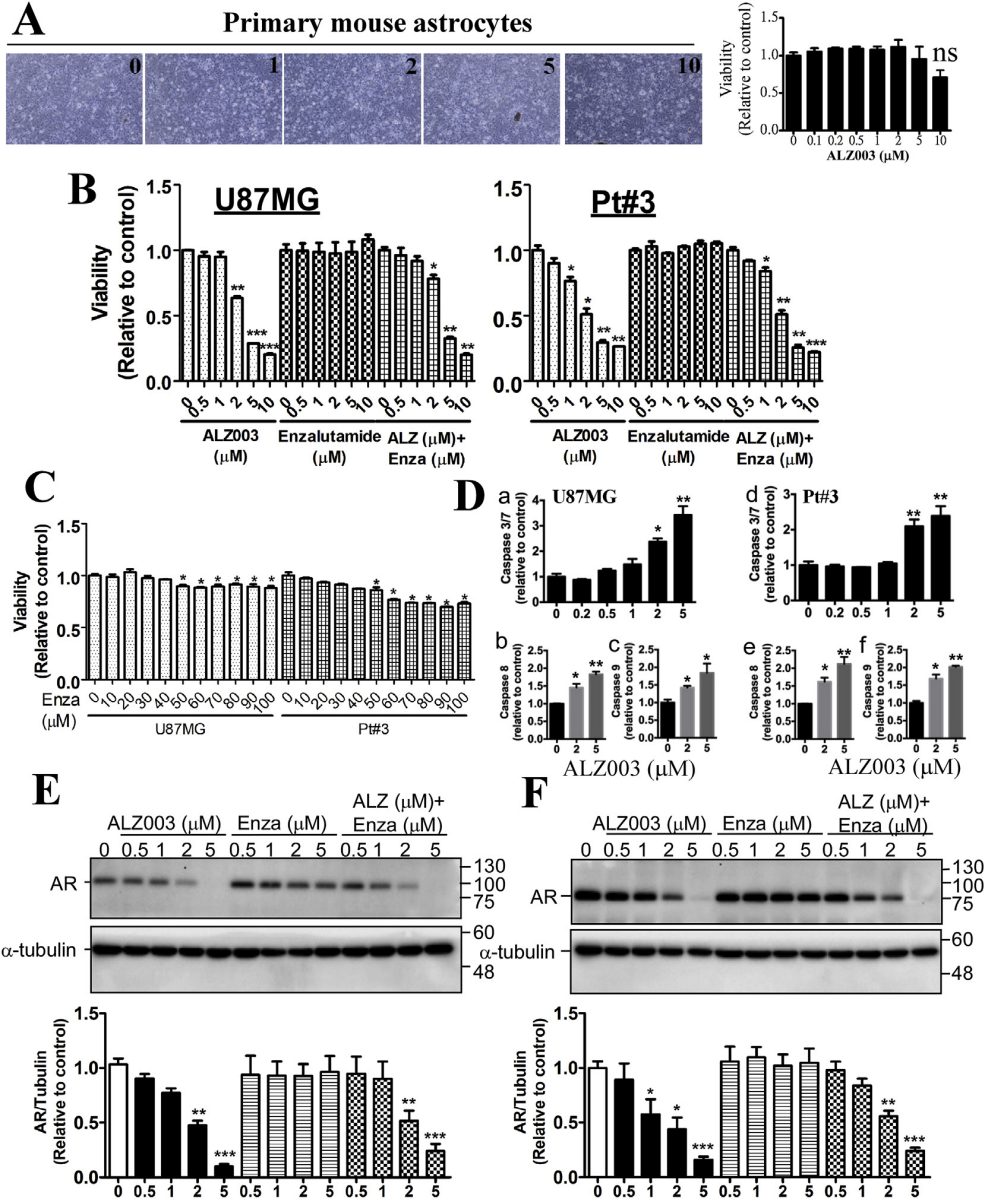
**Effect of ALZ003 on glioblastoma and AR expression.** A. Primary mouse astrocytes were treated with ALZ003 for 48 h, and then cell viability was determined by MTT assay. Left: representative images of astrocytes. Right: cell viability. Experiments were performed three times independently, and data were expressed as mean ± s.e.m. B, C. After treatment for 48 h, cell viability was estimated by MTT assay. Experiments were performed three times independently, and data were expressed as mean ± s.e.m. *P*-value was determined by Student's *t*-test. D. The cultured media from U87MG and Pt#3 with or without ALZ003 treatment for 48 h was analyzed by Caspase 3/7, 8 and 9-Glo activity kits. Experiments were performed three times independently, and data were expressed as mean ± s.e.m. (*p < 0.05, **p < 0.01). After treatment for 24 h, whole cell lysates of U87MG (E) or Pt#3 (F) were prepared and analyzed by western blotting using the anti-AR antibody. Lower panel is the quantitated results. Experiments were performed three times independently, and data were expressed as mean ± s.e.m. (*p < 0.05, **p < 0.01).

As AR expression is positively correlated with glioblastoma malignancy, we attempted to determine whether inhibition of AR is effective to kill glioblastoma. Based on the previous study showing that curcumin analogs, ALZ003 (also called ASC-JM17) and ASC-J9 are potent enhancer for AR protein degradation [15,20], we postulated that ALZ003 is potential for treating glioblastoma through the decrease of AR protein expression. Before clarifying that a novel compound is potent for treating diseases, the safety for normal cells has to be evaluated first. Therefore, we evaluated effect of ALZ003 on the survival of mouse astrocytes, TMZ-sensitive, and -resistant glioblastoma cells. In addition, since enzalutamide is a well-known AR inhibitor which is able to penetrate BBB [21], we attempted to compare the therapeutic effect of ALZ003 with enzalutamide. As the result, ALZ003 did not affect cell viability from 0.1 to 10 μM in normal astrocytes (Fig. 2A), whereas it significantly decreased cell proliferation of TMZ-sensitive, A172 and Pt#3, and -resistant glioblastoma cells, A172-R at ~2 μM (Supplementary Fig. S1A). However, enzalutamide failed to inhibit cell proliferation before 50 μM (Fig. 2B and C). Additionally, ALZ003 did not work synergistically with enzalutamide to inhibit proliferation (Fig. 2B). Further, until 50–100 μM, enzalutamide inhibited proliferation by only approximate 10–30% (Fig. 2C). These results indicate that ALZ003 is more potent than enzalutamide, and suggests that ALZ003 exhibits potent cytotoxicity targeting glioblastoma without toxic effect on normal brain cells. Moreover, proliferation of U87MG and U87MG-R was significantly inhibited by ALZ003 in time- and dose-dependent manners (Supplementary Fig. S1B). To elucidate whether ALZ003 induces apoptosis, we estimated levels of caspases 3/7, 8 and 9. Particularly, ALZ003 at 2 μM significantly increased activity of caspases 3/7, 8 and 9 in U87MG and Pt#3 cells (Fig. 2D), suggesting that ALZ003 activates both extrinsic and intrinsic apoptotic pathways. Importantly, AR protein, not mRNA, expression was dramatically decreased by ALZ003, not enzalutamide, treatment in a dose-dependent manner in TMZ-sensitive and -resistant cells (Fig. 2E and Supplementary Figs. S1C–D), suggesting that, in part, ALZ003 inhibits glioblastoma through decreasing the protein level of AR without affecting gene transcription of AR.

### 3.3 ALZ003 downregulates AR through inducing FBXL2-mediated ubiquitination

**Fig. 3:**
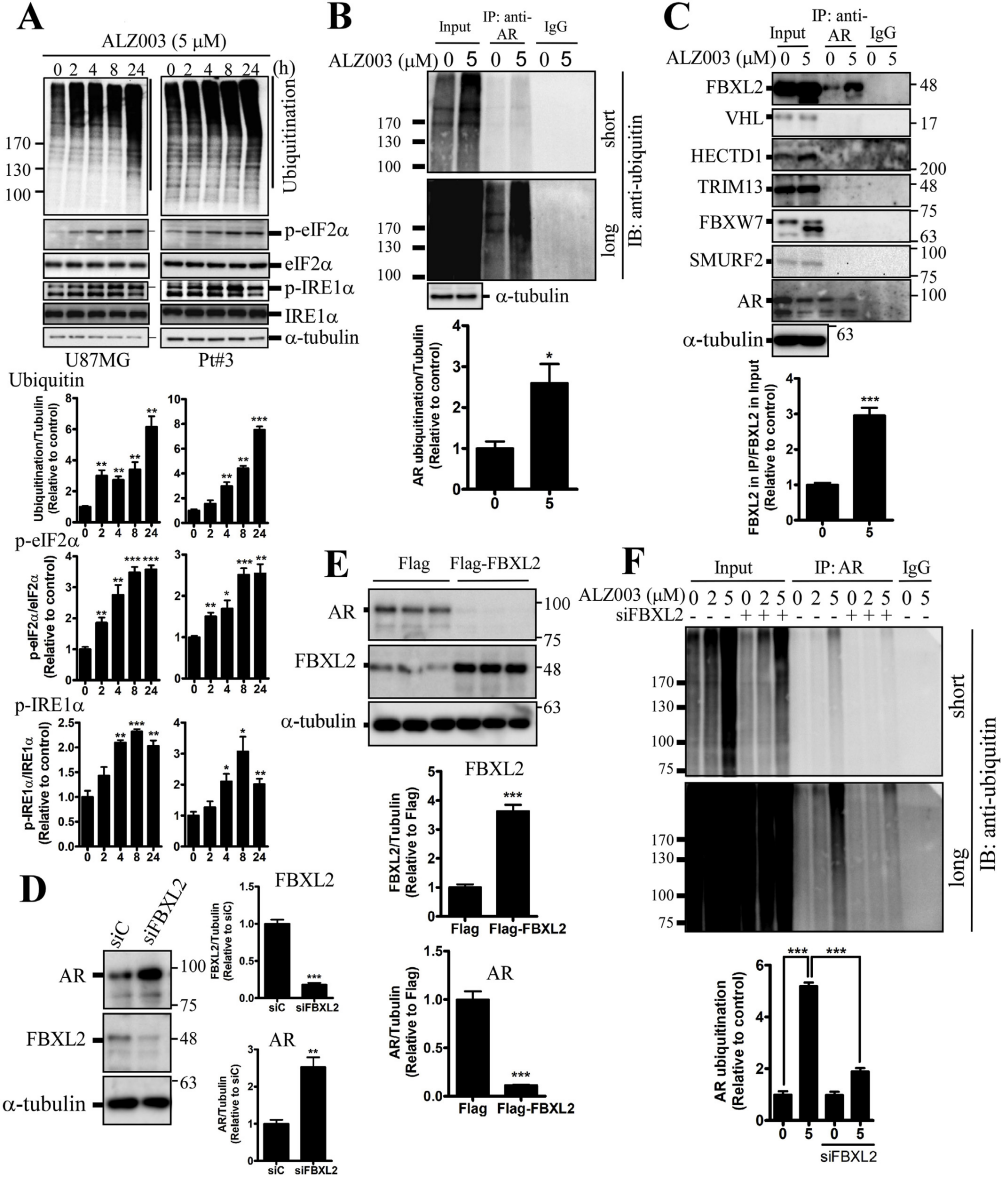
**FBXL2 plays a role in ALZ003-induced AR ubiquitination.** A. Effect of ALZ003 on protein ubiquitination and ER stress. After treatment for indicated time points, cell lysates were prepared and subjected to western blotting using the indicated antibody. Lower panel is the quantitated results. Experiments were performed three times independently, and data were expressed as mean ± s.e.m. *P*-value was determined by Student's *t*-test with the Control group. (*p < 0.05, **p < 0.01, ***p < 0.001). B. After treatment for 24 h, cell lysates of U87MG were immunoprecipitated using the anti-AR antibody, and the immune complex was analyzed by western blotting using the anti-ubiquitin antibody. Lower panel is the quantitated results, and the ubiquitin signal ranged from 100 kDa to the top of membrane was quantified. Experiments were performed three times independently, and data were expressed as mean ± s.e.m. (*p < 0.05). C. The association of AR with the ubiquitin E3 ligase. AR-precipitated immune complex was analyzed by western blotting. Experiments were performed three times independently, and data were expressed as mean ± s.e.m. (***p < 0.001). D. Effect of FBXL2 on AR expression. After transfection with Flag-FBXL2 for 24 h or FBXL2 knockdown (E) for 3 days (Left), AR expression was estimated. Experiments were performed three times independently, and data were expressed as mean ± s.e.m. (**p < 0.01, ***p < 0.001). E. After FBXL2 knockdown for 48 h, U87MG cells were treated with ALZ003 for 24 h. AR-precipitated immune complex was analyzed by western blotting using the anti-ubiquitin antibody. Experiments were performed three times independently, and data were expressed as mean ± s.e.m. (***p < 0.001).

ALZ003-mediated global protein ubiquitination was obviously increased in the time-dependent manner (Fig. 3A). Additionally, ALZ003 also triggered ER stress characterized by phosphorylated IRE1α and eIF2α (Fig. 3A). Furthermore, ALZ003 obviously induced AR ubiquitination in U87MG and Pt#3 cells confirmed by the immunoprecipitation assay using the anti-ubiquitin or anti-AR antibody (Fig. 3B and Supplementary Figs. S2A–B). To clarify the mechanism underlying AR ubiquitination, the interaction of AR with 6 tumor-suppressive E3 ligases [[22], [23], [24], [25], [26], [27]] including FBXL2, HECTD1, VHL, TRIM13, FBXW7 and SMURF2 were determined. Of that, the AR-FBXL2 interaction was detected after ALZ003 treatment (Fig. 3C). Particularly, knockdown of FBXL2 increased AR protein level (Fig. 3D), and overexpression of FBXL2 decreased AR protein level (Fig. 3E), suggesting that FBXL2 is responsible for ALZ003-induced AR protein degradation. Further, FBXL2 knockdown significantly attenuated ALZ003-induced AR ubiquitination (Fig. 3E), indicating that FBXL2 facilitates ALZ003-mediated AR ubiquitination. Importantly, FBXL2 knockdown significantly attenuated ALZ003-induced cytotoxicity (Supplementary Fig. S3), further suggesting that ALZ003 suppresses the growth of glioblastoma through inducing FBXL2-mediated AR ubiquitination.

### 3.4 ALZ003 induces lipid peroxidation and ROS accumulation

**Fig. 4:**
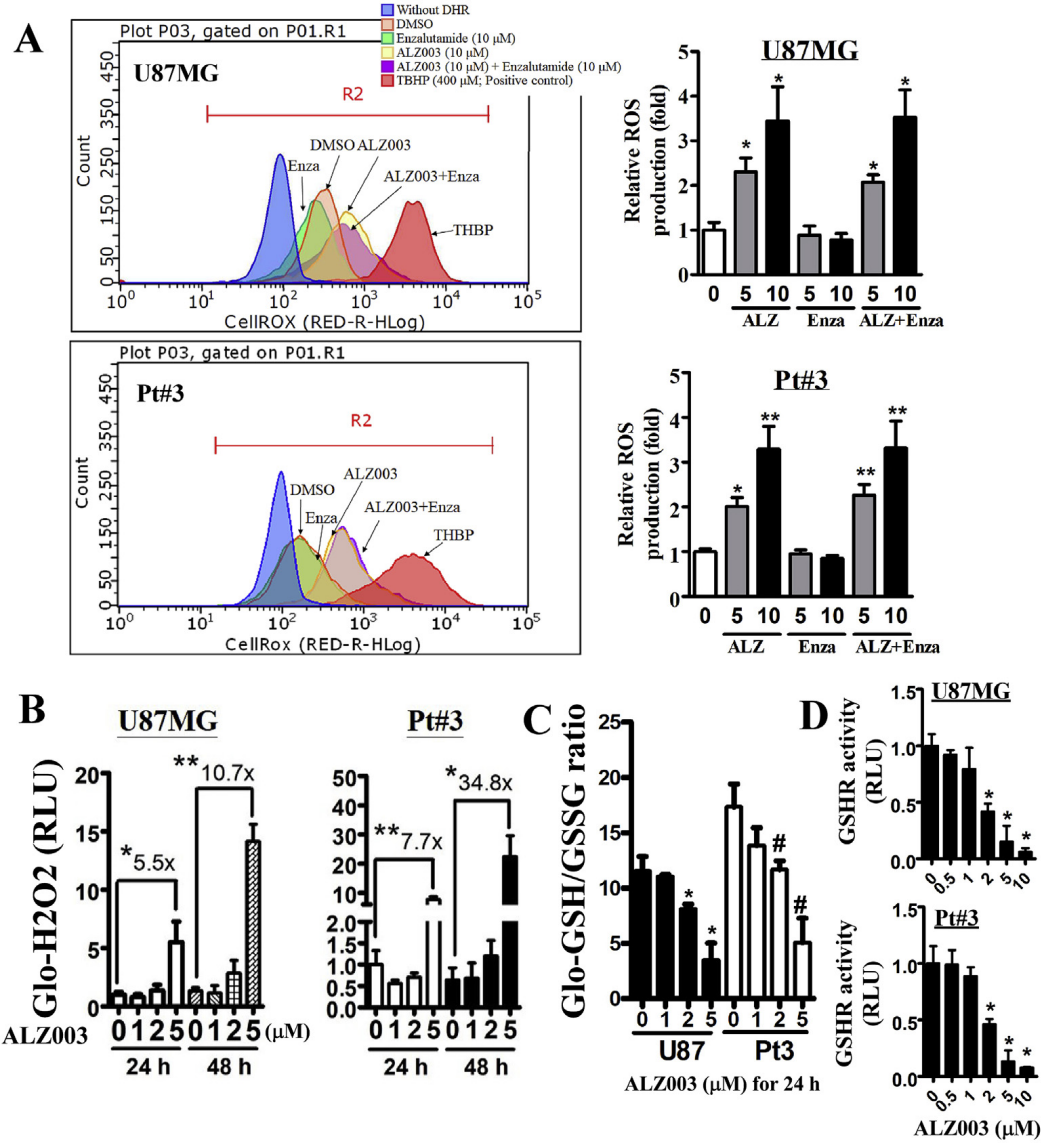
**ALZ003 increases ROS and lipid peroxidation through decreasing GPX4 expression.** A. After treatment for 24 h, U87MG cells were harvested for analyzing ROS production using DHR123. The number is the mean of fluorescence representing the level of ROS. Right panel is the quantitative result. Experiments were performed three times independently, and data were expressed as mean ± s.e.m. (*p < 0.05, **p < 0.01). Additionally, cells were analyzed for the H_2_O_2_ level (B), GSH/GSSG ratio (C) and GSHR activity (D). C. *p and ^#^*p* means the significant difference between control with treatment in U87MG and Pt#3, respectively. (*p, ^#^*p* < 0.05, **p < 0.01).

**Fig. 5:**
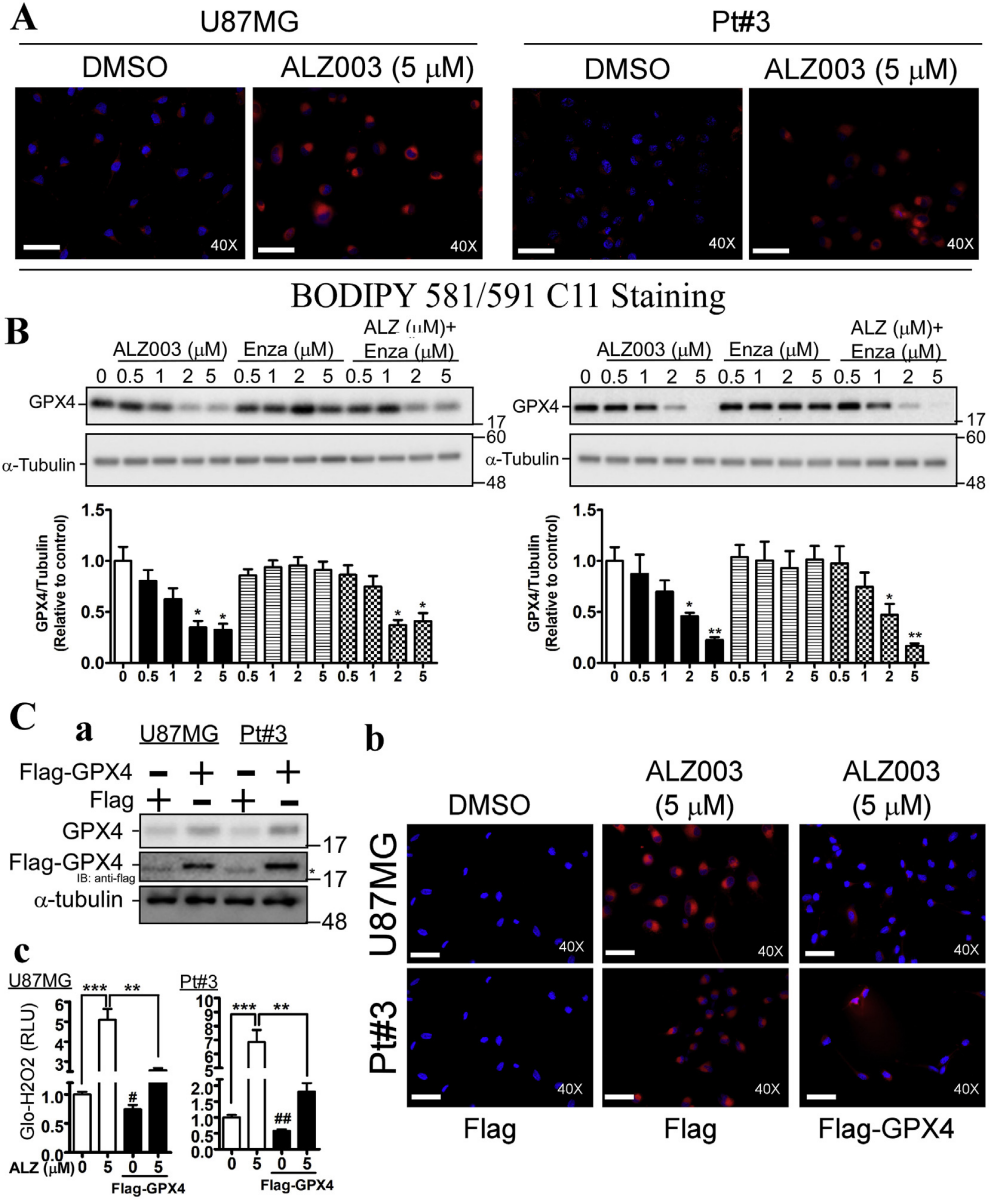
**ALZ003 induces lipid peroxidation through decreasing GPX4 expression.** A. After 24 h treatment, lipid peroxidation was estimated by staining cells with BODIPY 581/591C11 reagent. Immunofluorescent signal representing peroxidase lipid was photographed by fluorescent microscope under 40X magnification. The scale bar represented 0.1 mm. After treatment for 24 h, GPX4 expression in U87MG (B) and Pt#3 (C) cells was analyzed. Experiments were performed three times independently, and data were expressed as mean ± s.e.m. *P*-value was determined by Student's *t*-test compared with the Control group. (*p < 0.05, **p < 0.01). C. Effect of GPX4 on ALZ003-induced lipid peroxidation. (a). Overexpression of Flag-GPX4. *Star represents the non-specific band. After transfection for 24 h, cells were treated with ALZ003 for 24 h. Subsequently cells were subjected to BODIPY staining (b) and H_2_O_2_-Glo analysis (c) (**p < 0.01, ***p < 0.001; ^#^*p* < 0.05, ^##^*p* < 0.01 compared with the group without treatment and overexpression.) Immunofluorescent signal representing peroxidase lipid was photographed by fluorescent microscope under 40X magnification. The scale bar represented 0.1 mm.

Disturbance of ubiquitination homeostasis has been shown to induce ROS production or impair redox reaction [28,29]. Hence, we would like to evaluate whether ALZ003, which increases protein ubiquitination, affects the levels of ROS in glioblastoma. In comparison with enzalutamide, we found that ALZ003 significantly increased ROS production in U87MG and Pt#3 cells whereas enzalutamide did not exhibit any effects on inducing ROS (Fig. 4A and Supplementary Fig. S4A). This suggests that ROS production is majorly caused by ALZ003-induced degradation of AR and that inhibition of ligand-dependent activation of AR by the antagonist is not enough to impair redox homeostasis. In addition, ALZ003 significantly increased the H_2_O_2_ level in parallel with the reduction of GSH/GSSG ratio (Fig. 4B and C). Furthermore, glutathione reductase (GSHR) activity, which is responsible to maintain the level of reduced GSH, was significantly decreased by ALZ003 in a dose-dependent manner (Fig. 4D). Interestingly, ALZ003 potently induced lipid peroxidation (Fig. 5A) which is majorly caused by the deficiency of GPX4 expression [30], leading to ferroptosis [30]. Indeed, GPX4 expression was dramatically decreased after ALZ003 treatment in the dose-dependent manner in TMZ-sensitive and –resistant glioblastoma (Fig. 5B and Supplementary Fig. S4B), suggesting that ALZ003-mediated ROS accumulation is caused by the downregulation of GPX4. To further confirm this postulation, we estimated effect of ALZ003 on ROS in cells with or without Flag-GPX4 overexpression (Fig. 5C–a). We found that GPX4 overexpression obviously abolished ALZ003-induced lipid peroxidation (Fig. 5C–b) and hydrogen peroxide accumulation (Fig. 5C–c). These results indicate that ALZ003 is a strong inducer of oxidative stress in glioblastoma through inhibiting GPX4. Based on these evidence, tumor-suppressive effect of ALZ003 is mediated by both dysregulation of ubiquitination and accumulation of ROS in glioblastoma.

### 3.5 AR blocks ALZ003-impaired redox reaction through rescuing GPX4 expression

**Fig. 6:**
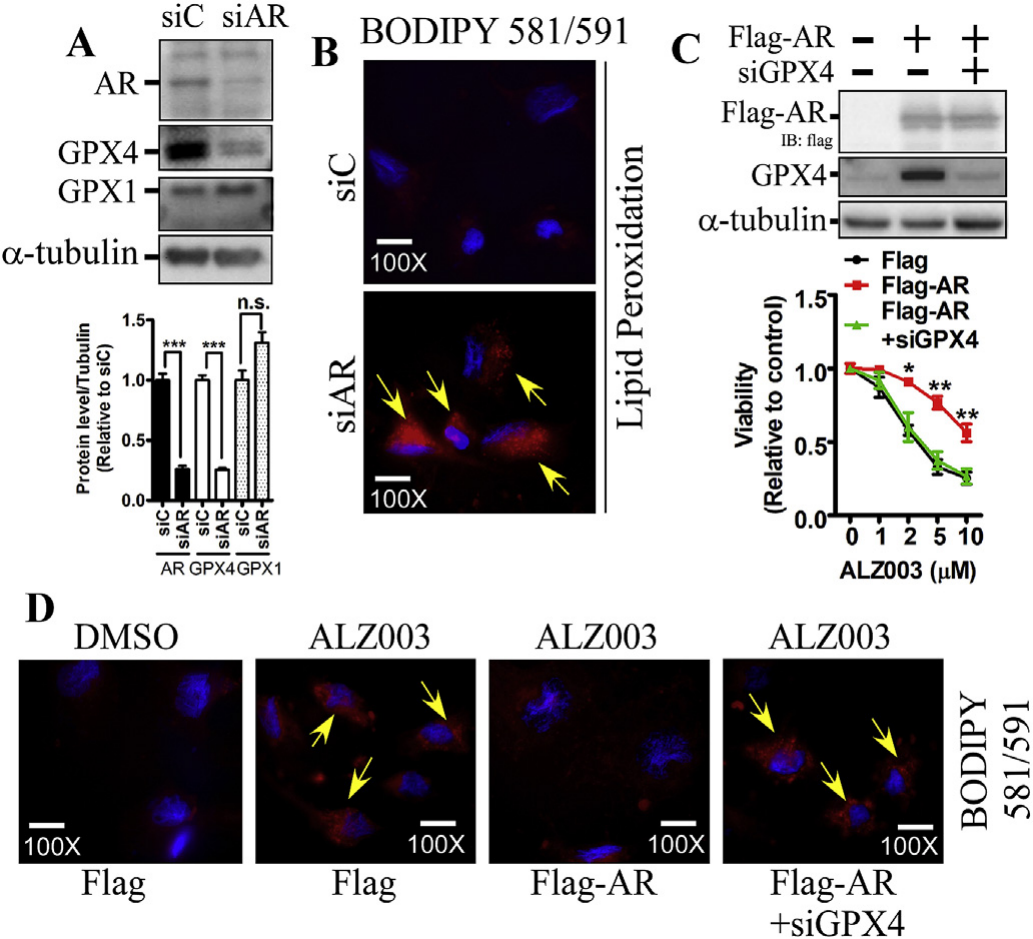
**AR regulates lipid peroxidation through GPX4.** A. Effect of AR knockdown on GPX4 expression. Lower panel is the quantitated results. Experiments were performed three times independently, and data were expressed as mean ± s.e.m. *P*-value was determined by Student's *t*-test. (***p < 0.001). B. AR knockdown induces strong lipid peroxidation photographed under 100X magnification in U87MG cells. Yellow arrows indicate lipid peroxidation. The scale bar represented 0.04 mm. C. After GPX4 knockdown for 2 days followed by transfection with Flag-AR for 1 day, U87MG cells were treated with 5 μM ALZ003 for 24 h. Viability was analyzed by MTT assay. Experiments were performed three times independently, and data were expressed as mean ± s.e.m. (*p < 0.05, **p < 0.01). Follow (C), lipid peroxidation was analyzed by BODIPY staining. (For interpretation of the references to colour in this figure legend, the reader is referred to the Web version of this article.)

Although ALZ003 decreased both AR and GPX4 expression, whether AR regulates GPX4 is still unknown. In U87MG cells, knockdown of AR obviously decreases the protein level of GPX4, not GPX1, and dramatically induced lipid peroxidation (Fig. 6A and B). This suggests that AR prevents lipid peroxidation through inducing GPX4 expression. Subsequently, we found that AR overexpression significantly attenuated ALZ003-induced cell death (Fig. 6C). However, in the presence of GPX4 knockdown, AR failed to induce the resistance to ALZ003, suggesting that ALZ003 inhibits glioblastoma through suppressing AR-mediated GPX4 expression. Additionally, AR overexpression obviously attenuated ALZ003-induced lipid peroxidation (Fig. 6D). The lipid peroxidation repressed by overexpression of AR was inhibited by the knockdown of GPX4. This further confirms that the ALZ003 inhibits glioblastoma through blocking AR-GPX4-regulated redox reaction, leading to lipid peroxidation and ROS accumulation.

### 3.6 ALZ003 inhibits the growth of glioblastoma without or with TMZ resistance *in vivo*

**Fig. 7:**
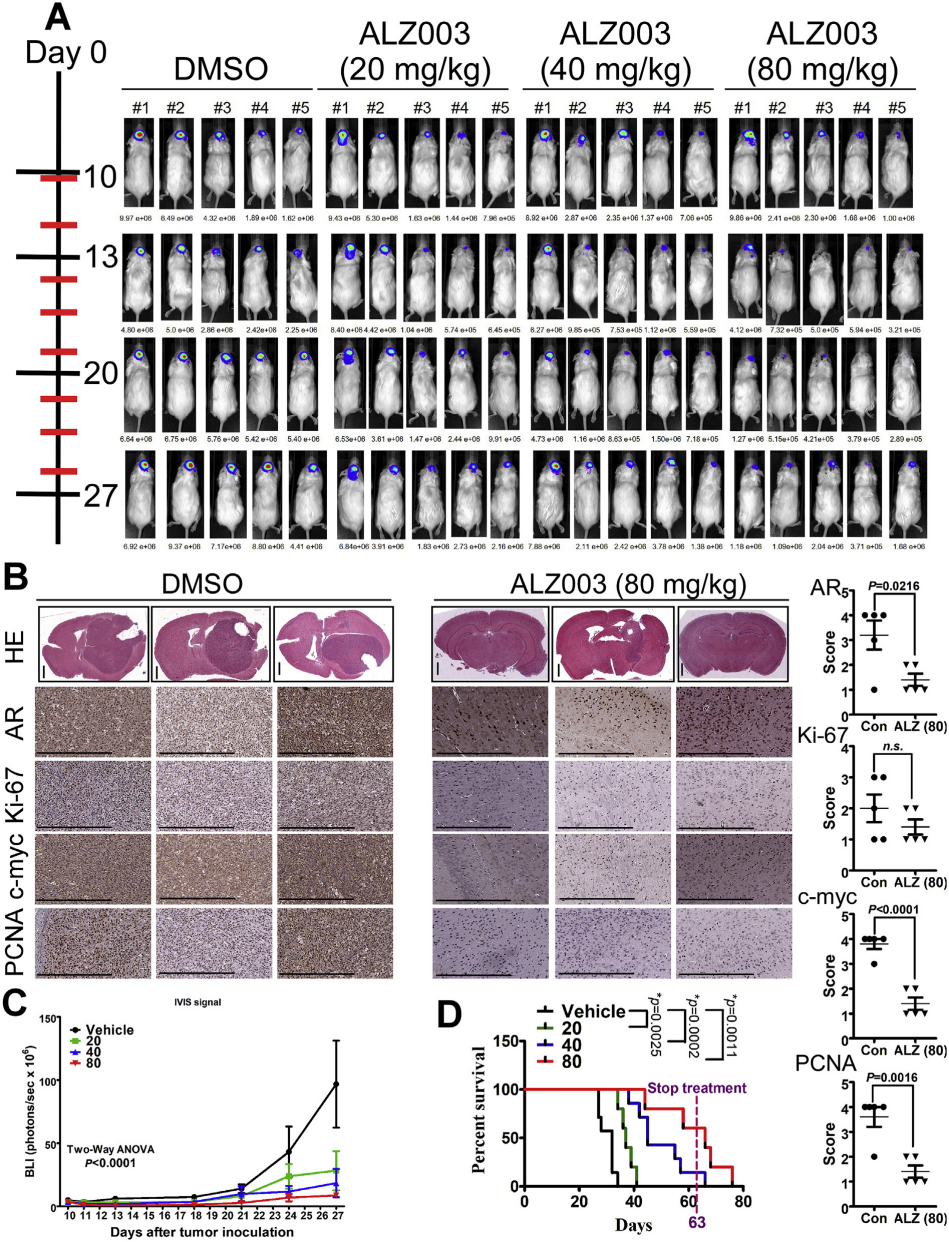
**Therapeutic effect of ALZ003 on the growth of glioblastoma *in vivo*.** A. Luciferase-expressed U87MG cells (2 × 10^5^) were intracranially transplanted into brains of nude mice. After 10 days, luciferase activity representing tumor size was monitored by IVIS200 system on Days 10, 13, 20 and 27. Experimental mice were administrated intravenously with ALZ003 three times per week. Left: The time line for IVIS200 evaluation (black line) and drug injection (red line). B. The mouse brain was paraffin embedded and subjected to histological analysis. Left panel: Brain slides were stained by hematoxylin and eosin or stained using indicated antibodies. Stained slides were photographed by microscope with 20X magnification. The scale bar for HE staining of whole brain slice is 1 mm, and that for other IHC images is 0.5 mm. Right: The IHC signal was quantitated by 4 Scores as described in *Materials and Methods*. *P*-value is indicated and “n.s.” means “not significant”. C. The statistical analysis for luciferase activity analyzed by two-way ANOVA (***p < 0.0001). D. The comparison of survival period was performed using Log-Rank Test. *P*-value for each paired comparison was indicated. ALZ003 administration was terminated on Day 63. (For interpretation of the references to colour in this figure legend, the reader is referred to the Web version of this article.)

**Fig. 8:**
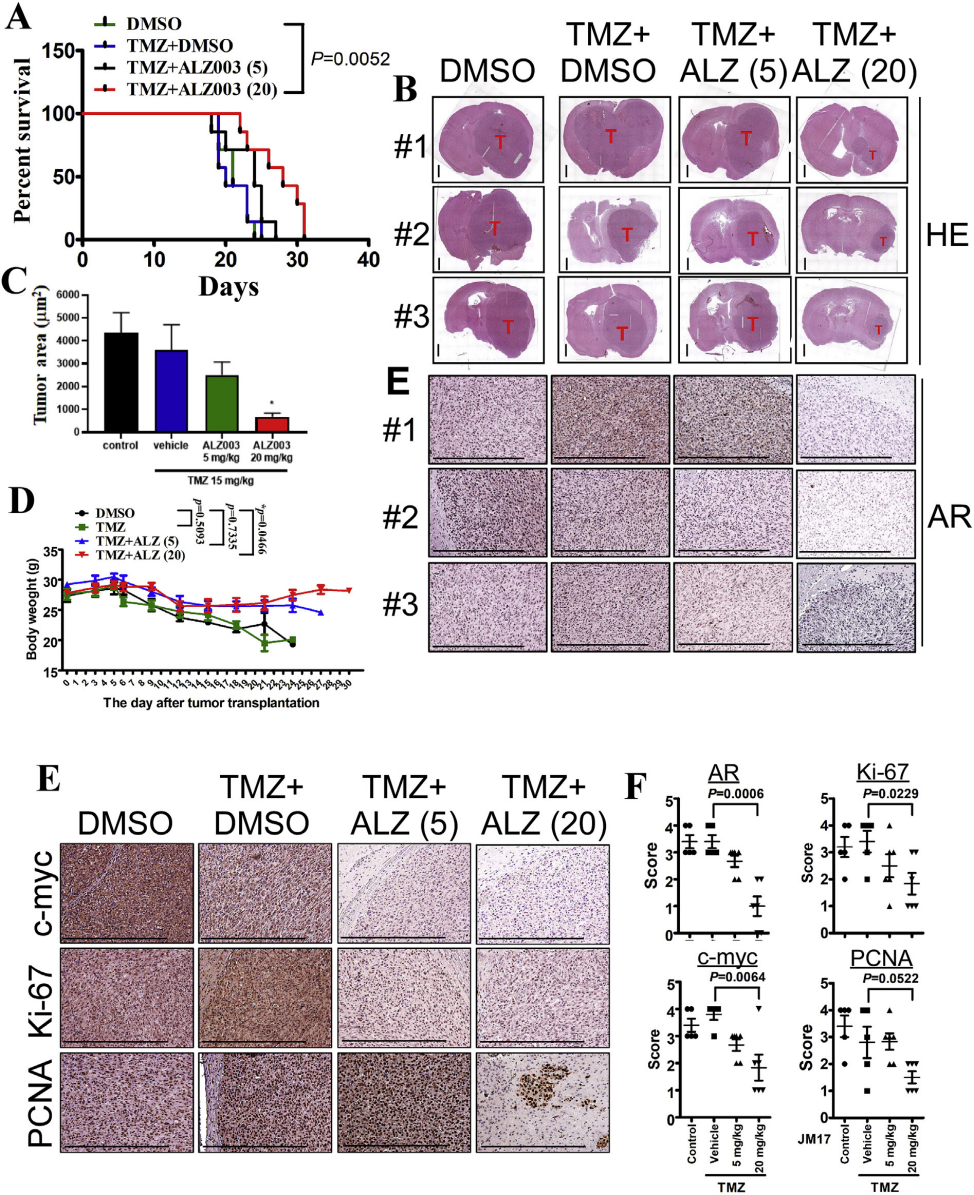
**Therapeutic effect of ALZ003 on the growth of TMZ-resistant glioblastoma *in vivo*.** A. After intracranial transplantation with TMZ-resistant U87MG cells for 10 days, mice were orally and intravenously administrated with TMZ (15 mg/kg) and ALZ003 three times per week, respectively. The date of death was recorded and analyzed by Log-Rank Test. (**p = 0.0052). B. The mouse brains were paraffin embedded and subject to slides preparation followed by hematoxylin- and eosin-staining. Tumor in each slide was indicated. The scale bar for HE staining of whole brain slice is 1 mm, and that for other IHC images is 0.5 mm. C. The area of tumor was quantitated by the Image J software. The difference with the control group was analyzed using Student's *t*-test (*p < 0.05). D. The alteration in body weight of transplanted mice during the process of experiment. The difference with DMSO group was analyzed using two-way ANOVA. *P*-value was indicated. E, F. The slides were immunostained using the antibody targeting AR, c-myc, Ki-67 or PCNA. The scale bar represents 0.5 mm. F. The intensity of the interested protein was quantified using Score definition. The comparison was performed using Student's *t*-test. *P*-value was indicated.

To clarify whether ALZ003 exhibits tumor-suppressive effect *in vivo*, we established a mouse model intracranially transplanted with glioblastoma cells. In Fig. 7, after transplantation with luciferase-expressed U87MG cells for 10 days, we started to monitor the alteration of luciferase activity representing tumor size. Importantly, intravenous administration of ALZ003 dramatically and significantly inhibited the growth of transplanted U87MG cells (Figs. 7A–C). In particular, mice received intracranial transplantation died on Days 27–34, whereas the survival period was significantly extended to Days 34–41, 38–66 and 44–76 by 20, 40 and 80 mg/kg of ALZ003 administration, respectively (Fig. 7D). Particularly, in addition to AR protein, other oncogenic markers, including c-myc, Ki-67 and PCNA, were also obviously decreased by ALZ003 administration confirmed by IHC staining **(Left Panel of** Fig. 7B) and H-Score quantitation (**Right Panel of** Fig. 7B). Additionally, we also evaluated whether ALZ003 inhibits TMZ-resistant glioblastoma *in vivo* **(**Fig. 8). The TMZ-resistant characteristics of U87MG-R were confirmed in our previous studies [8,18,19]. After transplantation with TMZ-resistant U87MG cells for 10 days, mice were orally and intravenously administrated with TMZ (15 mg/kg) and ALZ003, respectively. In Fig. 8A, tumor failed to respond to TMZ treatment due to its resistant phenotype. However, TMZ combined with 20 mg/kg ALZ003 significantly extended the survival period (Fig. 8A), and decreased tumor growth (Fig. 8B and C). Importantly, in contrast to DMSO-group of transplanted mice exhibiting obvious weight loss due to cachexia, the weight of experimental mice receiving ALZ003 treatment remained stable under intravenous administration of ALZ003 (Fig. 8D), suggesting that the dose for tumor-suppressive effect of ALZ003 is non-cytotoxicity for normal tissues. Furthermore, AR expression was obviously decreased in tumor area by ALZ003 administration (Fig. 8E). Under ALZ003 administration, oncogenic markers, including c-myc, Ki-67 and PCNA, were also obviously decreased in tumor area (Fig. 8F and G), indicating that ALZ003 is a potent and safe compound for glioblastoma therapy.

## 4 Discussion

Glioblastoma remains hard to be defeated by drug-mediated therapy because of very high frequency to develop drug resistance. In addition, most chemotherapeutic drugs, such as doxorubicin and cisplatin, are failed in brain tumor therapy due to the poor efficacy of crossing BBB [31,32]. Therefore, to identify a potent compound with BBB-crossing ability for treating glioblastoma is an urgent priority to benefit patients’ prognosis. This study demonstrated that ALZ003, a curcumin analog, exhibits highly potent tumor-suppressive effect on glioblastoma *in vitro* and *in vivo*. Through decreasing AR expression which regulates GPX4-mediated redox reaction, ALZ003 induced apoptosis and ferroptosis (Fig. 2, Fig. 5), characterized by caspase activity and lipid peroxidation, respectively, coupling with ROS accumulation. Particularly, we found that ALZ003 induces FBXL2-mediated AR ubiquitination, leading to downregulation of AR in glioblastoma. However, the mechanism to clarify how ALZ003 triggers ubiquitination targeting AR is still unknown, and this mechanism needs to be dissected in the future.

Herein, although we found that FBXL2-mediated ubiquitination induces AR degradation (Fig. 3 and Supplementary Fig. S2), the protein residues ubiquitinated by FBXL2 remain unknown. Many studies indicate that ubiquitination contributes to both AR degradation and AR transcriptional activity which is dependent on ubiquitinated residues [20,[33], [34], [35], [36]], and some ubiquitin E3 ligases targeting AR have been identified in prostate cancer. MDM2 and c-terminus of HSP70-interacting protein (CHIP)-mediated ubiquitination induces AR degradation in prostate cancer [20,37]. However, MDM2-mediated ubiquitination on lysing K311 contributes to enhance transcriptional activity of AR [35]. In addition, RNF6 promotes AR transcriptional activity through ubiquitinating residues K845/K847 [36]. These results suggest that different protein residues ubiquitinated by certain E3 ligases determine the fate of AR after ubiquitination.

Induction of AR degradation or repression of AR-mediated transcription is a well-known strategy to inhibit tumor development in prostate cancer [20,38]. However, whether a strategy targeting AR is effective for glioblastoma is unclear because of the difficulty in developing a drug with BBB-crossing ability. Curcumin has been shown to exhibit therapeutic effect on neurodegenerative diseases and brain tumor [10,12,13,39], indicating that curcumin is able to pass BBB to reach brain tissue. Additionally, curcumin inhibits multiple growth tumor through inhibiting AR expression [[40], [41], [42]]. However, the tumor-suppressive effect of curcumin *in vivo* was not always significant with that *in vitro* [14], suggesting the bioavailability of curcumin needs to be improved. ALZ003, a structurally analog to curcumin with superior stability, bioavailability, and potency, exhibits stronger anti-tumor effect on glioblastoma *in vitro* and *in vivo* through decreasing AR expression. The 48 h IC50 of ALZ003 in U87MG and Pt#3 is approximate <5 and 2 μM, respectively (Fig. 2), which is obviously lower than that of free curcumin (20–40 μM) [13,43], indicating that ALZ003 exhibit higher therapeutic effect. In particular, the mechanism of curcumin-mediated AR inhibition is unclear. To solve this question, we showed that ALZ003 decreased AR through inducing FBXL2-mediated ubiquitination (Fig. 3). Our results are consistent with other studies showing that another curcumin analog, ASC-J9, induces MDM2-mediated AR ubiquitination to inhibit prostate cancer [20], suggesting that AR is a critical player for anti-cancer effects of curcumin and its analogs.

Previously, we showed that DHEA, which is one kind of androgens or neurosteroids, induces TMZ resistance through enhancing DNA repair capacity [8]. We hypothesize that, in DHEA-enriched microenvironment, glioblastoma is more resistant in response to TMZ treatment through ligand-induced AR activation. The current study further emphasizes the promoting role of AR in growth and proliferation of glioblastoma, and indicates the disruption of AR functions by ALZ003 is potential to prevent DHEA-induced drug resistance. However, the positive effect of AR on tumor development is not restricted in ligand-induced AR. The studies on ligand-independent activation of AR in cancer is gradually increased in the recent years. Variant from of activated AR, AR-V7, has been shown to significant upregulation in tumor tissues, and shown to promote drug resistance [44]. In parallel, tyrosine phosphorylation is sufficient to maintain AR activity in the absence of androgens [6]. In the present study, effect of ALZ003 on ligand-independent AR activity remains unknown although we observed that ALZ003 decreased AR-V7 expression as well (data not shown).

Although AR expression is important for redox homeostasis [45], the role of AR in ferroptosis has never been mentioned. Classical ferroptosis is a characterized by peroxidation of polyunsaturated fatty acid-containing phospholipids, and is majorly caused by the inactivation of GPX4 [30]. Compared with GPX1 catalyzing hydrogen peroxide to water, the unique capacity of GPX4 is to detoxify hydroperoxides from peroxidized phospholipids [46]. Interestingly, ALZ003 induced GPX4 downregulation and lipid peroxidation simultaneously (Fig. 5), suggesting that ferroptosis is initiated by ALZ003. In addition, GPX4 overexpression prevented ALZ003-induced lipid peroxidation and ROS accumulation, suggesting that ALZ003-induced ferroptosis is mediated by GPX4. Particularly, GPX4 was positively regulated by AR, and overexpression of AR also prevented lipid peroxidation (Fig. 6), further confirming that ALZ003 induces ferroptosis through impairing AR-regulated GPX4 expression.

Based on our study, AR is a promising target in glioblastoma, driving us to study effect the inhibitor targeting AR, including BBB-penetrating enzalutamide. However, disruption of ligand-induced AR activation is probably not efficient to inhibit AR-mediated transcription due to ligand-independent activation [[47], [48], [49]]. Compared with enzalutamide which competes with androgen to prevent AR activation for gene expression, ALZ003 directly degrades AR, leading to much potently therapeutic efficacy. Therefore, a drug targeting AR for direct degradation is potential to be developed for treating glioblastoma. Herein, ALZ003 targeting AR for degradation strongly exhibits the therapeutic effect on glioblastoma, including TMZ-resistant tumor, *in vitro* and *in vivo*. In multiple glioblastoma cell lines, ALZ003 potently suppresses cell survival. Additionally, ALZ003 inhibits tumor growth and extends the survival period of mice received intracranial transplantation with glioblastoma cells. We conclude that the strategy targeting AR will be potential for treating glioblastoma, and ALZ003 will benefit patients’ life in the future.

## Declaration of competing interest

TCC, CHY and HC are employed by Allianz Pharmascience Ltd. when this collaborative study was conducted.
