# Supplementary material for: AR ubiquitination induced by the curcumin analog suppresses growth of temozolomide-resistant glioblastoma through disrupting GPX4-Mediated redox homeostasis
Source: Redox Biol. 2019 Dec 26;30:101413. doi: 10.1016/j.redox.2019.101413 (PMC6940696; doi:10.1016/j.redox.2019.101413)
Supplement: Multimedia component 1 [file mmc1.docx]

**Supplementary Materials**

**AR Ubiquitination Induced by the Curcumin Analog Suppresses Growth of Temozolomide-Resistant Glioblastoma through Disrupting GPX4-Mediated Redox Homeostasis**

Tzu-Chi Chen^1#^, Jian-Ying Chuang^2,3,4,5#^, Chiung-Yuan Ko^2,3,4,5^, Tzu-Jen Kao^2,3,4^, Pei-Yu Yang^2,3,4^, Chun-Hui Yu^1^, Ming-Sheng Liu^6^, Siou-Lian Hu^2,3,4^, Yu-Ting Tsai^7^, Hardy Chan^1^, Wen-Chang Chang^4,7*^, Tsung-I Hsu^2,3,4,5*^

^1^Allianz Pharmascience Limited, Taipei, Taiwan.

^2^Graduate Institute of Neural Regenerative Medicine, College of Medical Science and Technology, Taipei Medical University, Taipei, Taiwan.

^3^Ph.D. Program for Neural Regenerative Medicine, College of Medical Science and Technology, Taipei Medical University and National Health Research Institutes, Taipei, Taiwan.

^4^TMU Research Center of Neuroscience, Taipei Medical University, Taipei, Taiwan.

^5^TMU Research Center of Cancer Translational Medicine, Taipei Medical University, Taipei, Taiwan.

^6^National Institute of Cancer Research, National Health Research Institutes, Taiwan.

^7^Graduate Institute of Medical Sciences, College of Medicine, Taipei Medical University, Taipei, Taiwan

**
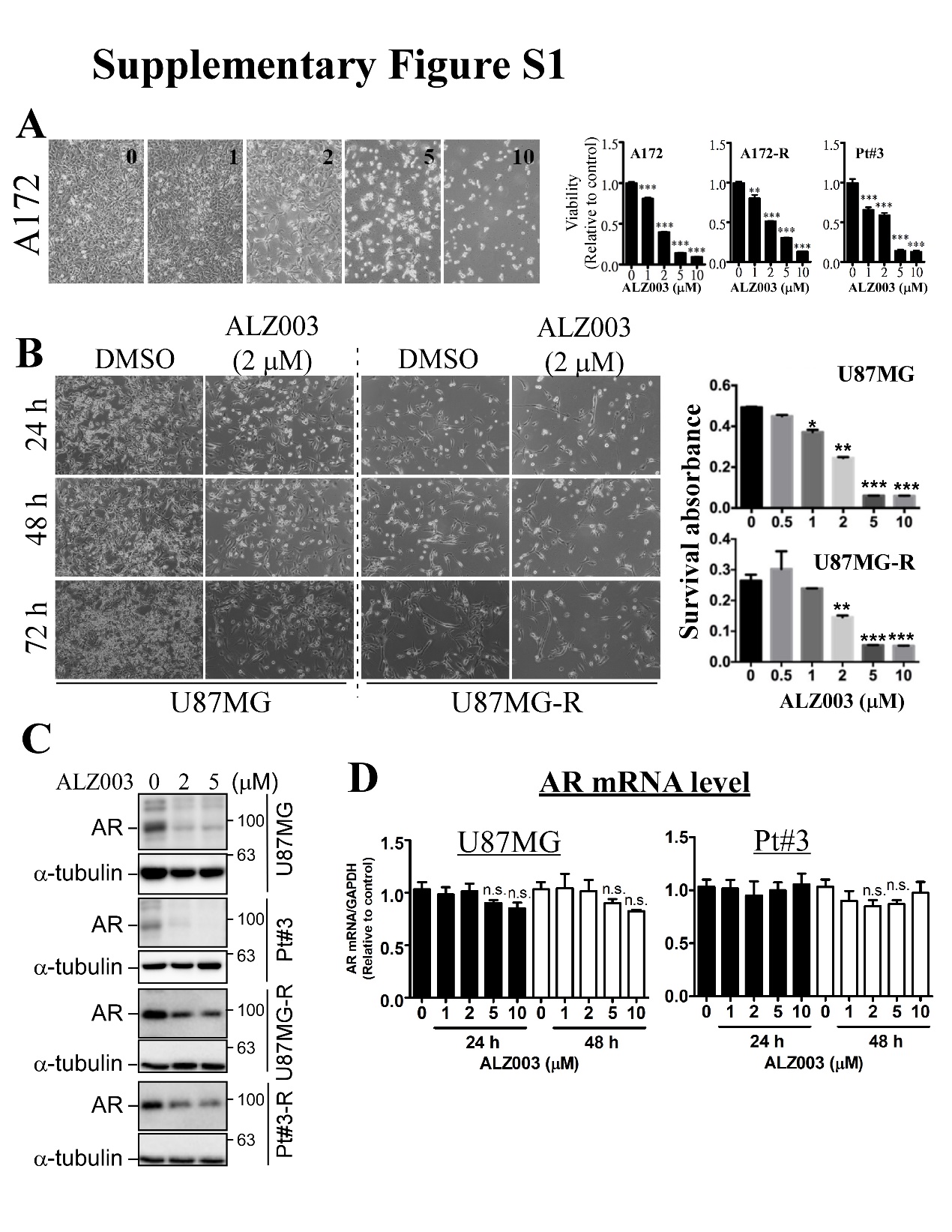
Supplementary Figure S1. Effect of ALZ003 on proliferation and AR expression in glioblastoma.** A. After treatment for 48h, TMZ-sensitive A172 and Pt#3, and –resistant A172-R cells were harvested for MTT assay. B. Left: After treatment with 2 μM ALZ003 for 24, 48 and 72h, U87MG and U87MG-R cells were photographed. Right: After treatment with multiple doses of ALZ003 for 48 h, cells were harvested for MTT assay. (**p*<0.05, ***p*<0.01, ****p*<0.001). C. After treatment for 24h, cells were harvested and protein lysates were prepared for western blotting using indicated antibodies. D. After treatment, extracted RNA was subjected to reverse transcription followed by qPCR.

**
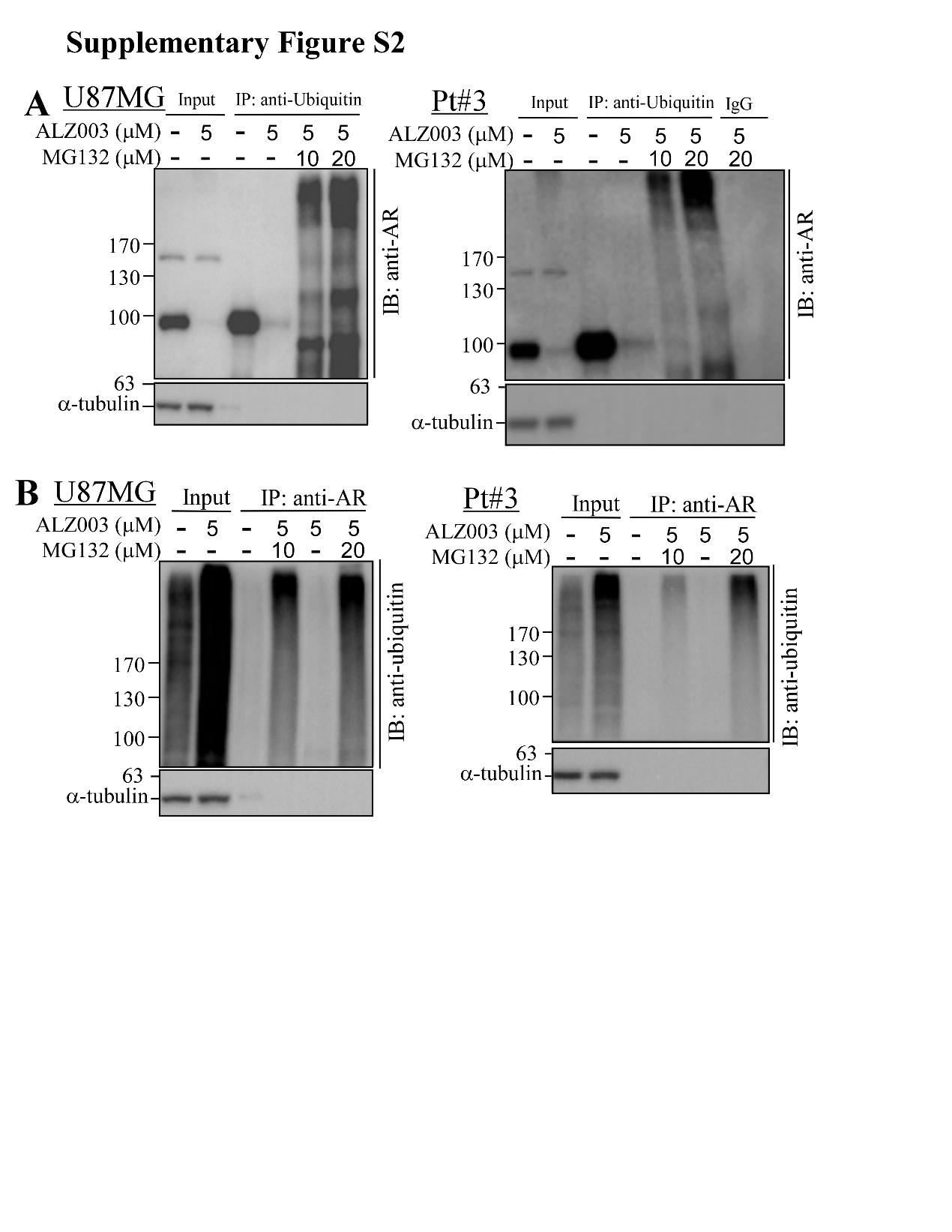
**

**Supplementary Figure S2. Effect of ALZ003 on AR ubiquitination in glioblastoma.** After treatment for 24h in the presence or absence of MG132, cells were harvested in RIPA lysis buffer. Protein lysates were subjected to immunoprecipitation by the indicated antibody. Immuno-complex was analyzed by western blotting using indicated antibodies. A, IP: anti-Ubiquitin antibody; IB: anti-AR antibody; B: IP: anti-AR antibody; IB: anti-Ubiquitin antibody

**
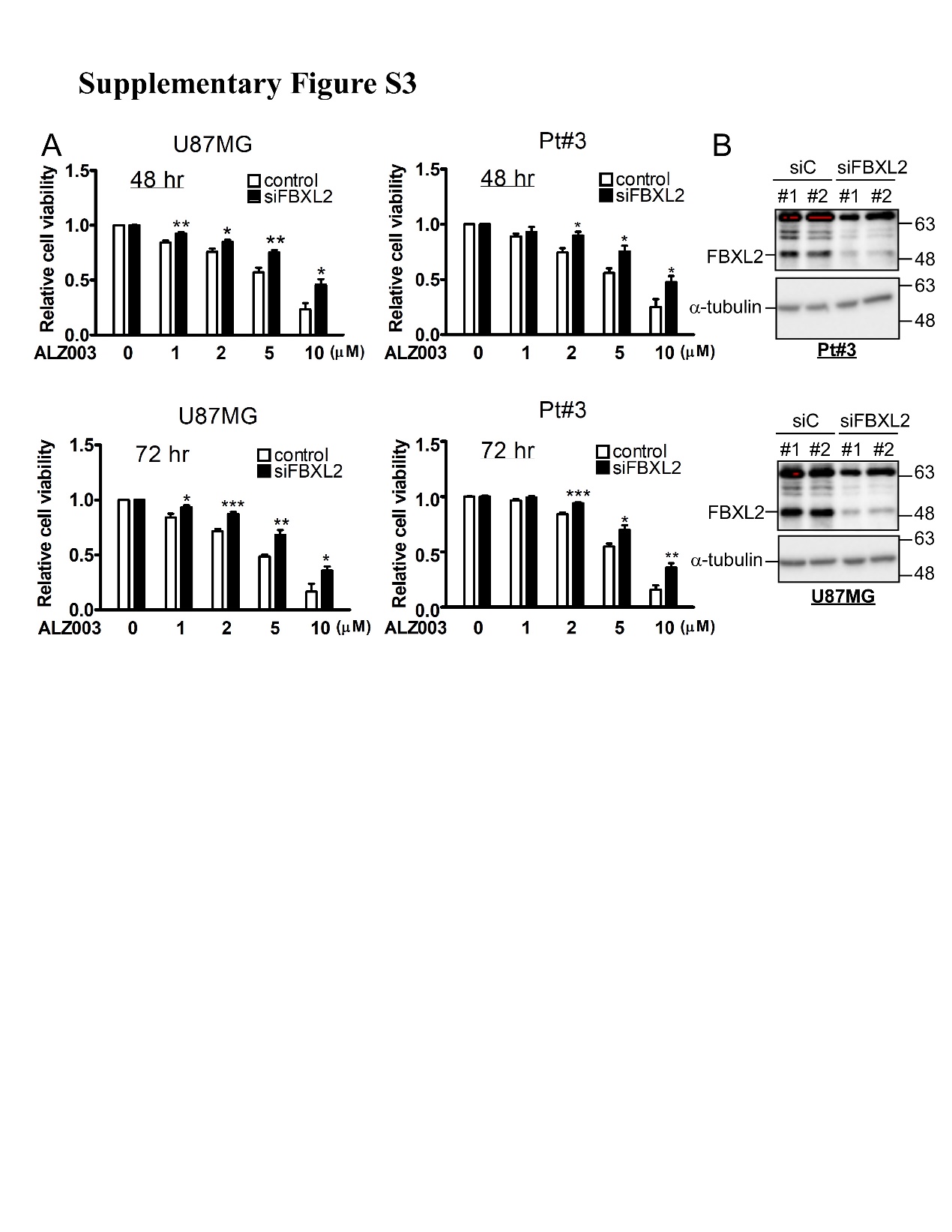
**

**Supplementary Figure S3. Effect of FBXL2 knockdown on ALZ003-reduced proliferation in glioblastoma.** A. After FBXL2 knockdown for 72h, cells were treated with ALZ003. Viability was estimated by MTT assay. Experiments were performed three times independently, and data were expressed as mean±s.e.m. *P*-value between Control group and siFBXL2 group was determined by Student’s *t* test. B. Cell lysates were subjected to confirm FBXL2 knockdown using western blotting.

**
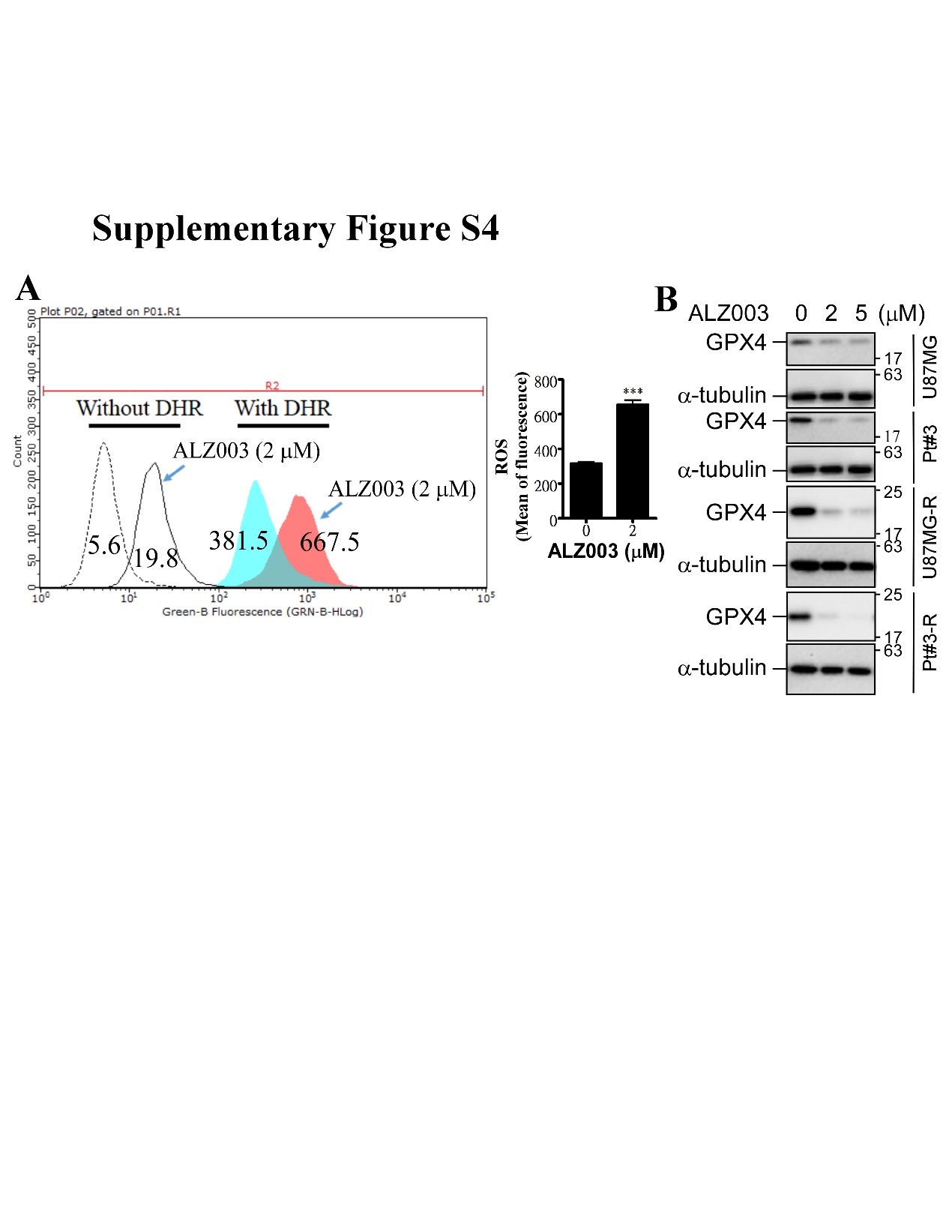
**

**Supplementary Figure S4. Effect of ALZ003 on redox homeostasis and GPX4 expression in glioblastoma.** A. After treatment for 24h, U87MG cells were harvested for analyzing ROS production using DHR123. The number is the mean of fluorescence representing the level of ROS. Right panel is the quantitative result. Experiments were performed three times independently, and data were expressed as mean±s.e.m. (****p*<0.001). B. After treatment for 24h, GPX4 expression was analyzed using western blotting.

| **Brain (n)** | **GBM (n)** | **Reference** | **Fold (GBM/N)** | ***p*-value** |
| --- | --- | --- | --- | --- |
| 4 | 27 | Bredel et al. 2005. | **2.495** | **1.05E-09** |
| 23 | 81 | Sun et al. 2006. | **2.12** | **7.13E-17** |
| 10 | 542 | TCGA Brain | **1.744** | **1.05E-06** |
| 2 | 29 | Liang et al. 2005.. | 3.085 | 0.121 |
| 7 | 27 | Shai et al. 2003. | 1.107 | 0.067 |
| 3 | 22 | Lee et al. 2006. | -1.681 | 1 |
| 4 | 80 | Murant et al. 2008. | **1.438** | **1.58E-07** |

**Supplementary Table S1. The comparison of AR expression in glioma with normal brain tissue using the *Oncomine* website**

| **Antibody** | **Brand** | **Titer** |
| --- | --- | --- |
| Androgen receptor (AR) | abcam (Cambridge, UK) | WB: 1:1000  IHC: 1:100  IP: 1 μg |
| α-tubulin | MilliporeSigma Corporate (St. Louis, MO, USA) | WB: 1:15000 |
| Flag-tag | MilliporeSigma Corporate | WB: 1:5000 |
| Ubiquitin  Ubiquitin | GeneTex International  Corporation (HsinChu, Taiwan)  MilliporeSigma Corporate | WB: 1:3000  IP: 1 μg |
| p-eIF2α | Cell Signaling Technology ( Danvers, MA, USA) | WB: 1:1000 |
| eIF2α | Cell Signaling Technology | WB: 1:1000 |
| p-IRE1α | abcam | WB: 1:1000 |
| IRE1α | Cell Signaling Technology | WB: 1:1000 |
| VHL | GeneTex International  Corporation | WB: 1:1000 |
| HECTD1 | GeneTex International  Corporation | WB: 1:1000 |
| TRIM13 | abcam | WB: 1:1000 |
| FBXW7 | GeneTex International  Corporation | WB: 1:1000 |
| SMURF2 | GeneTex International  Corporation | WB: 1:1000 |
| GPX4 | GeneTex International  Corporation | WB: 1:1000 |
| GPX1 | GeneTex International  Corporation | WB: 1:1000 |
| c-myc | Cell Signaling Technology | IHC: 1:300 |
| Ki-67 | Cell Signaling Technology | IHC: 1:300 |
| PCNA | Cell Signaling Technology | IHC: 1:300 |

**Supplementary Table S2. Used antibody in this study.**
